# Supplementary material for: Priority Setting in the Polish Health Care System According to Patients’ Perspective
Source: Int J Environ Res Public Health. 2021 Jan 28;18(3):1178. doi: 10.3390/ijerph18031178 (PMC7908543; doi:10.3390/ijerph18031178)
Supplement: Supplementary file 1 [file ijerph-18-01178-s001.pdf]

Priority setting in the Polish Health Care System

- patients perspective

Dear Sir / Madam,

We would appreciate if you took part in a scientific study aimed at defining the direction of changes necessary to increase the efficiency of a healthcare system. The research is being conducted by the Healthcare Policy Chair at the Medical University of Lodz and the Research Centre for Healthcare Strategy and Health Protection Policy at the Warsaw School of Economics in cooperation with the National Medical University in Kiev, and it seeks to promote optimal solutions in the terms of organization, functioning and financing of healthcare. By completing this questionnaire form, you agree to give your consent to use the introduced data strictly for research purposes, and the authors of the study guarantee you anonymity.

Research Team

DEMOGRAPHIC QUESTIONS

Gender F ☐ M ☐

Age *(the year of birth)* ☐

Education

Primary ☐

Secondary ☐

University ☐

Residence

Village/rural area ☐

Small town (<20 000 residents) ☐

Medium-sized city (20 000-100 000 residents) ☐

Big-sized city (100 000-500 000 residents) ☐

Biggest city (> 500 000 residents) ☐

Married

Yes ☐

No ☐

Employment

Student ☐

Employed ☐

Unemployed ☐

What is your marital status? *(-3- definitely bad, -2 bad, -1 – rather bad, 0 – no opinion, 1 rather good, 2 – good, 3 – definitely good)*

-3☐ -2☐ -1☐ 0☐ 1☐ 2☐ 3☐

How do you assess your health status? *(-3- definitely bad, -2 bad, -1 – rather bad, 0 – no opinion, 1 rather good, 2 – good, 3 – definitely good)*

-3☐ -2☐ -1☐ 0☐ 1☐ 2☐ 3☐

Are you interested in healthcare system changes? *(-3- definitely not, -2 not, -1 – rather not, 0 – no opinion, 1 rather yes, 2 – yes, 3 – definitely yes)*

-3☐ -2☐ -1☐ 0☐ 1☐ 2☐ 3☐

Frequency of receiving health care

a few times a month ☐

once a month ☐

every three months ☐

every six months ☐

once a year ☐

less frequently than once a year ☐

Form of health care the respondents use most frequently

Basic health care ☐

Specialist care in outpatient clinics ☐

Hospitals ☐

Form of health care the respondents use more frequently

Public/State facilities ☐

Private facilities ☐

<u>POLISH HEALTH CARE SYSTEM</u>

1. General assessment of the health care system in Poland *(-3- definitely bad, -2 bad, -1 – rather bad, 0 – no opinion, 1 rather good, 2 – good, 3 – definitely good)*

-3☐ -2☐ -1☐ 0☐ 1☐ 2☐ 3☐

2. Factors contributing to delayed diagnosis *(-3- definitely not, -2 not, -1 – rather not, 0 – no opinion, 1 rather yes, 2 – yes, 3 – definitely yes)*

visiting primary care physicians too late

3☐ -2☐ -1☐ 0☐ 1☐ 2☐ 3☐

visiting medical specialists too late -3☐ -2☐ -1☐ 0☐ 1☐ 2☐ 3☐

too long waiting times for medical specialists -3☐ -2☐ -1☐ 0☐ 1☐ 2☐ 3☐

too long waiting times for hospital treatment -3☐ -2☐ -1☐ 0☐ 1☐ 2☐ 3☐

poorly developed prevention efforts -3☐ -2☐ -1☐ 0☐ 1☐ 2☐ 3☐

insufficient financial resources allocated to health care services -3☐ -2☐ -1☐ 0☐ 1☐ 2☐ 3☐

ignoring the disease symptoms by patients -3☐ -2☐ -1☐ 0☐ 1☐ 2☐ 3☐

self-healing -3☐ -2☐ -1☐ 0☐ 1☐ 2☐ 3☐

too late diagnosis -3☐ -2☐ -1☐ 0☐ 1☐ 2☐ 3☐

poor availability of diagnostic tests -3☐ -2☐ -1☐ 0☐ 1☐ 2☐ 3☐

too long waiting times for diagnostic test results -3☐ -2☐ -1☐ 0☐ 1☐ 2☐ 3☐

not taking care of health by patients -3☐ -2☐ -1☐ 0☐ 1☐ 2☐ 3☐

3. To what extent do you agree with the following opinions on the public health care system *(-3- definitely not, -2 not, -1 – rather not, 0 – no opinion, 1 rather yes, 2 – yes, 3 – definitely yes)*

patients are treated with kindness and care -3☐ -2☐ -1☐ 0☐ 1☐ 2☐ 3☐

it is easy to make an appointment with a primary care doctor -3☐ -2☐ -1☐ 0☐ 1☐ 2☐ 3☐

it is easy to obtain information on availability of health services -3☐ -2☐ -1☐ 0☐ 1☐ 2☐ 3☐

medical treatment is entirely free -3☐ -2☐ -1☐ 0☐ 1☐ 2☐ 3☐

treatment conditions are good -3☐ -2☐ -1☐ 0☐ 1☐ 2☐ 3☐

doctors are willing to give referrals to medical specialists if the patient's condition requires so -3☐ -2☐ -1☐ 0☐ 1☐ 2☐ 3☐

patients can expect immediate medical assistance -3☐ -2☐ -1☐ 0☐ 1☐ 2☐ 3☐

all patients are treated equally -3☐ -2☐ -1☐ 0☐ 1☐ 2☐ 3☐

4. Evaluation of the quality of health services: *(-3- definitely bad, -2 bad, -1 – rather bad, 0 – no opinion, 1 rather good, 2 – good, 3 – definitely good)*

in public institutions of the health care system -3☐ -2☐ -1☐ 0☐ 1☐ 2☐ 3☐

in private institutions of the health care system -3☐ -2☐ -1☐ 0☐ 1☐ 2☐ 3☐

5. Evaluation of the availability of health services: *(-3- definitely bad, -2 bad, -1 – rather bad, 0 – no opinion, 1 rather good, 2 – good, 3 – definitely good)*

in public institutions of the health care system -3☐ -2☐ -1☐ 0☐ 1☐ 2☐ 3☐

in private institutions of the health care system -3☐ -2☐ -1☐ 0☐ 1☐ 2☐ 3☐

6. Factors influencing the effectiveness of the health care system *(-3- definitely not, -2 not, -1 – rather not, 0 – no opinion, 1 rather yes, 2 – yes, 3 – definitely yes)*

organization of the health care system -3☐ -2☐ -1☐ 0☐ 1☐ 2☐ 3☐

financing of health care -3☐ -2☐ -1☐ 0☐ 1☐ 2☐ 3☐

number of practicing doctors -3☐ -2☐ -1☐ 0☐ 1☐ 2☐ 3☐

competences of practicing doctors -3☐ -2☐ -1☐ 0☐ 1☐ 2☐ 3☐

hospital infrastructure -3☐ -2☐ -1☐ 0☐ 1☐ 2☐ 3☐

medical equipment in diagnostics and therapy -3☐ -2☐ -1☐ 0☐ 1☐ 2☐ 3☐

costs of medications -3☐ -2☐ -1☐ 0☐ 1☐ 2☐ 3☐

prevention/ health education	-3☐ -2☐ -1☐ 0☐ 1☐ 2☐ 3☐

7. Factors/aspects/areas requiring immediate improvements *(-3- definitely not, -2 not, -1 – rather not, 0 – no opinion, 1 rather yes, 2 – yes, 3 – definitely yes)*

quality of health care	-3☐ -2☐ -1☐ 0☐ 1☐ 2☐ 3☐
availability of health care	-3☐ -2☐ -1☐ 0☐ 1☐ 2☐ 3☐
range of benefits financed from health insurance contributions	-3☐ -2☐ -1☐ 0☐ 1☐ 2☐ 3☐
financing of health care	-3☐ -2☐ -1☐ 0☐ 1☐ 2☐ 3☐
infrastructure of health care entities (building, equipment)	-3☐ -2☐ -1☐ 0☐ 1☐ 2☐ 3☐
knowledge, skills and competences of medical personnel	-3☐ -2☐ -1☐ 0☐ 1☐ 2☐ 3☐

8. Sources of financing health care services *(select max. 3 items)*

**regarding the following items**:	the state (taxes)	compulsory health insurance	voluntary health insurance	the patient's own financial resources
primary healtcare	☐	☐	☐	☐
specialist care	☐	☐	☐	☐
highly specialized services	☐	☐	☐	☐
hospital treatment	☐	☐	☐	☐
prevention services	☐	☐	☐	☐
dental treatment	☐	☐	☐	☐
medical rehabilitation	☐	☐	☐	☐
hospice and palliative care	☐	☐	☐	☐
emergency medical services	☐	☐	☐	☐
spa treatment	☐	☐	☐	☐

9. Healthcare professional salaries – opinion about minimum and maximum remunerations

	**min**	**max**
doctors	PLN	PLN
nurses	PLN	PLN
paramedics	PLN	PLN

10. Which of the areas of healthcare should most funds be allocated to ? *(max 3 items*

primary healtcare ☐

specialist care ☐

highly specialized services ☐

hospital treatment ☐

prevention services ☐

dental treatment ☐

rehabilitation ☐

hospice and palliative care ☐

emergency medical services ☐

spa treatment ☐

11. Should patients pay for every medical treatment?

no ☐

yes (*give the maximum contribution)* ☐ PLN

12. Factors/areas requiring changes in the Polish health care system *(-3- definitely not, -2 not, -1 – rather not, 0 – not opinion, 1 rather yes, 2 – yes, 3 – definitely yes)*

system of training medical personnel	-3☐	-2☐	-1☐	0☐	1☐	2☐	3☐
financing health services	-3☐	-2☐	-1☐	0☐	1☐	2☐	3☐
limited role of the state in the decision-making process in the system	-3☐	-2☐	-1☐	0☐	1☐	2☐	3☐
role of health insurance	-3☐	-2☐	-1☐	0☐	1☐	2☐	3☐
free-market rules	-3☐	-2☐	-1☐	0☐	1☐	2☐	3☐
priorities in the health care system	-3☐	-2☐	-1☐	0☐	1☐	2☐	3☐
